# Impact of manual toothbrush design on plaque removal efficacy

**DOI:** 10.1186/s12903-023-03518-6

**Published:** 2023-10-25

**Authors:** Alyson Axe, Wolf Dieter Mueller, Helen Rafferty, Tomas Lang, Peter Gaengler

**Affiliations:** 1 Haleon (formerly GSK Consumer Healthcare), Weybridge, Surrey UK; 2 ORMED Institute for Oral Medicine, University of Witten/Herdecke, Witten, Germany

**Keywords:** Toothbrushing, Dental plaque, Filaments, Manual toothbrush

## Abstract

**Background:**

Effective dental plaque removal is essential for oral health. Different toothbrush parameters including head-size, filament-diameter and interdent-height and different brushing movements like horizontal, rotating and vertical may affect plaque removal efficacy. The purpose of the study was to examine plaque removal efficacy of different design parameters of manual toothbrushes.

**Methods:**

Eight manual toothbrushes were tested using a validated robot test to examine efficacy of toothbrush on replicated human teeth. Characteristics tested were: (i) head-size, (ii) filament-diameter, (iii) cutting-height, (iv) hardness, (v) interdental-height. Each test ran five times in horizontal, rotating, vertical movements. Simulated Plaque removal was evaluated using automated plaque planimetry: 30 fields/tooth, 13 areas representing buccal, lingual, proximal tooth sites. The Kolmogorov-Smirnov-test was applied to test tooth surface variables for normal distribution of plaque removal values. Parameters were analysed by independent two-sample t-test to assess mean differences. Where null hypothesis of normality was rejected, the Wilcoxon-Mann-Whitney-U-test was used.

**Results:**

Plaque removal was significantly better with toothbrush having smaller head-size (compact vs. full-size); smaller filament-diameter (0.12 mm vs. 0.15 mm); larger cutting-height (12 mm vs. 9 mm); softer filaments (0.15 or 0.18 mm vs. 0.23 mm) and greater interdent-height difference (8.5/11 mm vs. 10/11 mm).

**Conclusions:**

Manual brushes allowing filaments free to flex with longer, softer and/or having a difference in filament length overall removed significantly more simulated plaque as compared to more standard flat trim, stiff brushes with shorter, harder bristles and a larger head size. While limited by the in vitro nature of the study design, this indicates that the advances in toothbrush design can further enhance plaque removal.

## Background

Dental plaque is one of the etiologic or predisposing factors for development of dental caries and plaque-associated periodontal diseases [1]. Mechanical removal of plaque through toothbrushing is considered to be generally recommended to avoid plaque-associated diseases or tooth decay [2, 3] .

Effective plaque removal depends on various factors like adequate brushing time, brushing technique, force of brushing, etc. that varies from individual to individual. Toothbrush innovations try to compensate for inadequate brushing time and technique by creating toothbrushes with an increased plaque removal design compared to the conventional flat trimmed toothbrushes created in the early 20th century [4]. These advancements have given rise to brushes with differing parameters including different bristle amounts, arrangements, lengths and diameter, varying head designs and lengths [4–6].

Numerous studies have been conducted to determine the plaque removal efficacy of manual toothbrush parameters [7]. Both clinical and in vitro testing have yielded results indicating that differing bristle configuration, such as criss-cross and angled bristles, impacts plaque removal [4]. A clinical study showed that a toothbrush with softer, tapered, cross-angled bristles was more efficacious at plaque removal compared to a toothbrush with standard (medium) bristles [6]. In other studies, criss-cross bristle configurations have proved advantageous compared to standard straight bristles, showing effective plaque removal from the smooth surfaces of teeth as well as gingival margin and interpproximal surfaces [8]. Furthermore, a meta review of clinical data showed that angled bristles are more efficacious than standard flat trimmed brushes [9]. In contrast, a systematic review found little to no difference between tapered or end-rounded filaments concerning to plaque removal efficacy [1].

While clinical testing yield valuable results when measuring plaque removal by toothbrushes, laboratory testing of toothbrush efficacy for simulated plaque removal using a calibrated and clinically validated robot simulation model allows results to be reproducible through standardisation of brushing movement, force and time [10].

In this current study, a validated robot simulation model was used to assess the simulated plaque removal efficacy of different toothbrush designs using three different brushing movements, vertical, horizontal and rotating movements. The design parameters tested included variations in head-size, cutting-height, hardness, filament diameter and interdental-height difference.

## Methods

**Fig. 1:**
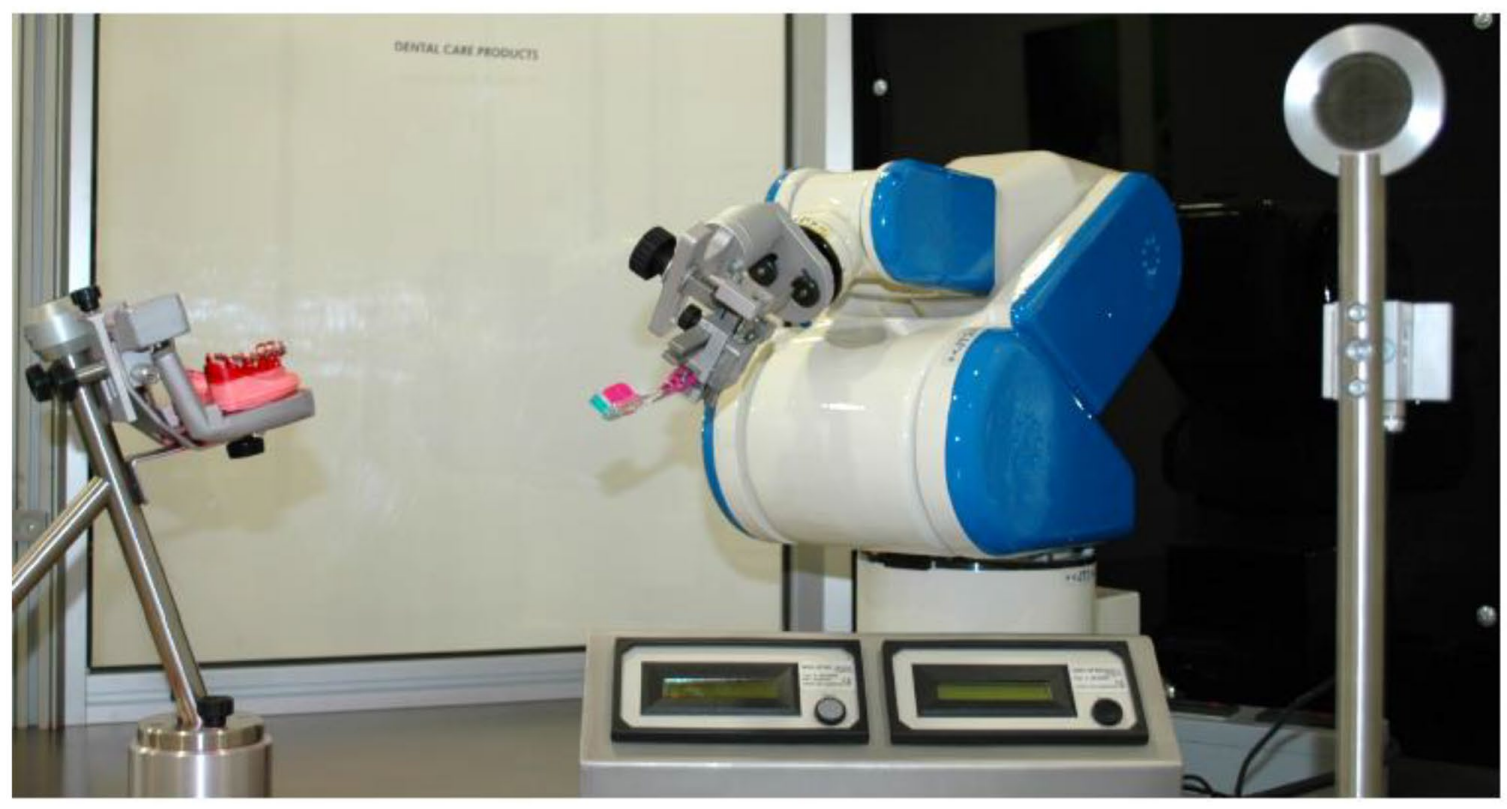
Robot set-up toothbrushing set up. *Adapted from* [10], *Creative Commons Attribution 4.0 International Licence*

This study was carried out at a Germany-based research facility. The 6-axis Robot FS002N (Kawasaki Robotics, Akashi, Hyogo, Japan) was programmed to repeat the most commonly observed brushing movements of uninstructed individuals, horizontal (amplitude 5 mm), vertical (10 mm) and rotating (diameter 10 mm) [11] individually in series, with five runs for each movement at a brushing force of 3.5 N (Fig. 1) [12]. This allowed the robot to undertake a clinically validated in vitro test [10, 12] to assess the effects of bristle configuration on KaVo™ (KaVo, Biberach, Germany) artificial plastic teeth. These are replicated from natural human dentition and included four incisors, one canine, two premolars and three molars placed in anatomical positions within a mounting plate to represent mandibular human dentition. The teeth were covered in simulated plaque, designed to mimic the adherent properties of natural plaque, consisting of a specialized red, inorganic formula to help assess plaque removal [10].

**Fig. 2:**
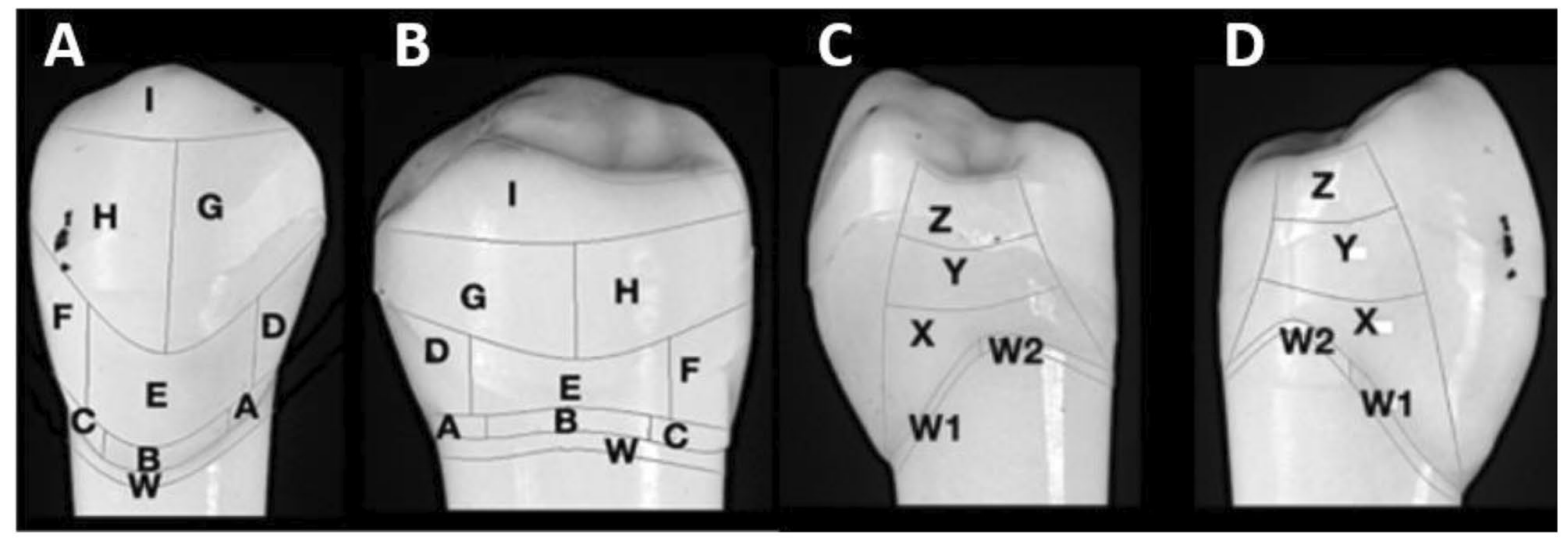
Seven variables of tooth surfaces. Automated plaque detection fields. **(A)** Buccal sites (towards the cheek); **(B)** Lingual sites (towards the tongue); **(C)** Mesial sites (proximal in-between teeth, anterior site); **(D)** Distal sites (proximal in-between teeth, posterior side); ABCDF: Risk fields near the gum line and approximately between the teeth; Total: Total mean plaque reduction at all 30 tooth sites

Simulated plaque removal was calculated at 30 planimetrical fields by computer-assisted optical automatic plaque planimetry (APP) [Fig. 2]. This works by rotating each test tooth in front of a high definition focusing analysis camera for computer assisted processing. Mean simulated plaque reduction was ascertained by measuring simulated plaque levels pre- and post-brushing at seven variables of tooth surfaces: all buccal tooth sites [Fig. 2A]; all lingual tooth sites [Fig. 2B]; buccal and, separately, lingual risk fields near the gum line and interpproximly between the teeth (ABCDF fields; Fig. 2AB); all mesial sites [Fig. 2 C]; all distal sites [Fig. 2D] and total mean simulated plaque reduction at all 30 planimetrical fields [Fig. 2A–D]. The ‘W’ fields, which usually sit below the gum line, were analysed and included in the ‘all sites’ assessments but are not presented separately [13].

**Fig. 3:**
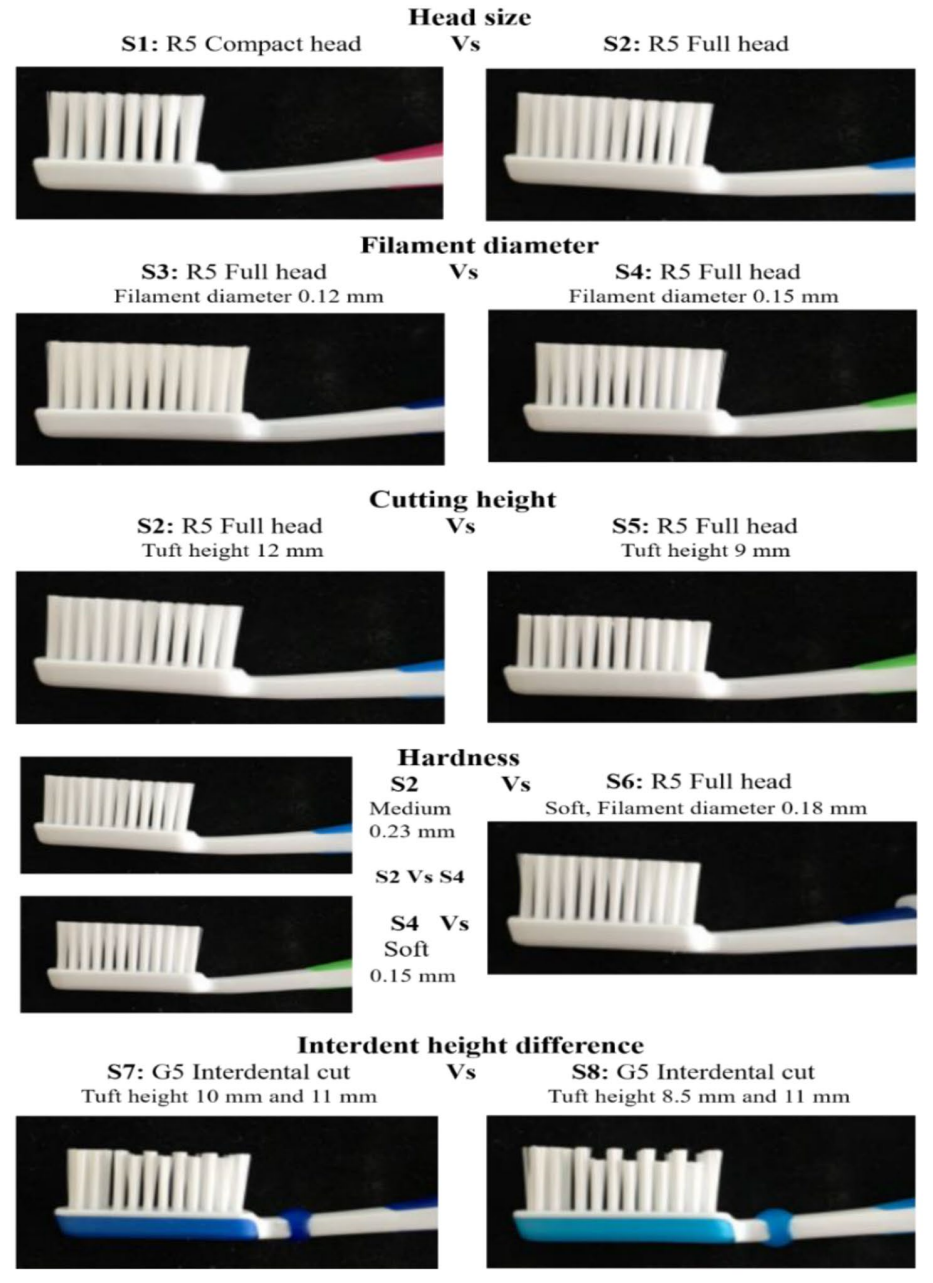
Toothbrushes tested, and comparisons made. *Eight Dr Best® toothbrushes, Haleon (Formerly GSK Consumer Healthcare, Brentford, UK) with differing properties were used*

Eight Dr Best® toothbrushes, Haleon (formerly GSK Consumer Healthcare, Brentford, UK) with end-rounded nylon filaments, differing properties were used (Fig. 3); only the characteristics relevant to each comparison mentioned-below, which were different are noted here (e.g., tuft height was only noted when this characteristic was relevant to the comparison). These toothbrushes were named- S1: R5 Compact Head; S2: R5 Full Head, Medium hard: 12 mm tuft height, 0.23 mm: filament diameter; S3: R5 Full Head, Extra Soft, 0.12 mm filament diameter; S4: R5 Full Head, Soft, 0.15 mm filament diameter; S5: R5 Full Head, Medium hard, 9 mm tuft height; S6: R5 Full Head, Soft; 0.18 mm filament diameter; S7: G5 Interdental cut, tuft heights 10 and 11 mm; S8: G5 Interdental cut, tuft heights 8.5 and 11 mm.

The following parameters were tested to assess cleaning simulated plaque removal efficacy of the differing configurations: Head-Size (S1 vs. S2); Filament-Diameter (S3 vs. S4); Tuft Cutting-Height (S5 vs. S2); Hardness (S2 vs. S4; S2 vs. S6; S4 vs. S6) and Interdental Tuft-Height Difference (S7 vs. S8). The comparisons and characteristics of interest are detailed in Fig. 3.

### Statistical testing

Statistical testing of the data was completed by the ORMED Institute using IBM SPSS Statistics Premium, release 24.

The Kolmogorov-Smirnov-test (K-S-test; one sample test) was applied to test the 13 variables of tooth surfaces- buccal, lingual, mesial, distal, buccal risk fields ABCDF, lingual risk fields ABCDF, buccal and lingual root field W, mesial and distal root fields W1 and W2 and total for a normal distribution of simulated plaque removal values. Significance level α = 0.10 (10%) was used to test this important assumption of parametric t-test. As a result, the null hypothesis (H0) of normality was clearly rejected for 4 of the 13 parameters/ surfaces (buccal, lingual, buccal W and total).

The consequence is that 9 of the 13 parameters were analysed by independent two samples t-test. Each single toothbrush was tested against each other. On the other hand, the four non-parametric parameters were analysed by Wilcoxon-Mann-Whitney-U-test (WMW-test or U-test). WMW- test can be applied on ordinal or also unknown distributions – contrary to the t-test – and it is nearly as efficient as the parametric t-test (power efficiency (pe) of the WMW-test: 95% > pe > 90%). Application of t-test in this study was combined with the Levene test for testing the null hypothesis that the variances of the tested toothbrushes were equal. Where the Levene’s test did not show significance, the ‘normal’ version of the t-test was used. Where Levene’s test showed significance, the t-test with a correction term was used. The significance level for Levene’s test was set at α = 0.10 (10%). For all two-tailed tests of differences in cleaning efficacy between brushes, the significance level was set at p-value of α = 0.05 (5%).

## Results

The numerical values for simulated plaque- removal with different toothbrush designs from the tooth surfaces are represented in Table 1.

**Table 1:** Statistical analysis of cleaning efficacy (% plaque removal)

Head Size									
	Horizontal			Rotating			Vertical		
	S1	S2	p-value	S1	S2	p-value	S1	S2	p-value
**Buccal**	95.1	94.37	NS	92.95	94.76	S2*	74.52	80.83	S2**
**ABCDF Buccal**	69.23	61.16	S1*	95.2	95.81	NS	90.63	94.12	S2**
**Lingual**	96.04	95.96	NS	69.79	60.5	S2*	65.72	67.99	S2*
**ABCDF Lingual**	83.81	77.8	S1*	60.8	58.79	NS	57.26	58.3	S2***
**Mesial**	77.51	69.28	S1*	59.23	58.91	S1*	63.37	74.72	NS
**Distal**	72.85	68.74	NS	76.49	77.44	NS	73.98	79.17	NS
**Total**	82.42	77.89	S1*	76.06	74.4	S1**	70.91	75.86	S2*
**Filament Diameter**									
	**Horizontal**			**Rotating**			**Vertical**		
	**S3**	**S4**	**p-value**	**S3**	**S4**	**p-value**	**S3**	**S4**	**p-value**
**Buccal**	97.23	96.62	NS	97.41	96.73	S3*	82.83	79.46	NS
**ABCDF Buccal**	82.14	72.61	S3**	74.34	72.96	NS	72.02	74.27	NS
**Lingual**	97.93	97.5	S3*	97.18	97.32	NS	94.31	93.11	NS
**ABCDF Lingual**	92.44	86.58	S3***	87.68	86.19	NS	79.53	80.85	NS
**Mesial**	91.97	76.5	S3***	84.3	72.96	S3***	67.87	68.74	NS
**Distal**	87.9	74.72	S3***	78.73	66.32	S3***	65.66	58.98	S3*
**Total**	86.75	75.66	S3**	79.98	72.63	S3*	69.59	68.67	NS
**Cutting Height**									
	**Horizontal**			**Rotating**			**Vertical**		
	**S5**	**S2**	**p-value**	**S5**	**S2**	**p-value**	**S5**	**S2**	**p-value**
**Buccal**	80.15	94.37	S2**	94.69	94.76	NS	92.94	80.83	S5**
**ABCDF Buccal**	28.89	61.16	S2***	53.89	58.91	S2*	45.13	74.72	S2***
**Lingual**	87.94	95.96	S2**	96.2	95.81	NS	95.18	94.12	S5*
**ABCDF Lingual**	53.21	77.8	S2***	76.34	77.44	S2***	69.96	79.17	NS
**Mesial**	50.79	69.28	S2**	56.42	60.5	NS	49.01	67.99	S2***
**Distal**	46.83	68.74	S2**	54.36	58.79	S2*	42.06	58.3	S2***
**Total**	47.22	68.11	S2**	57.96	62.33	S2*	51.57	67.94	S2*
**Interdent Height**									
	**Horizontal**			**Rotating**			**Vertical**		
	**S7**	**S8**	**p-value**	**S7**	**S8**	**p-value**	**S7**	**S8**	**p-value**
**Buccal**	85.32	80.03	S7**	88.04	96.54	S8*	76.94	95.39	S8*
**ABCDF Buccal**	46.94	68.05	S8***	53.64	75.32	S8***	63.35	70.37	S8**
**Lingual**	90.48	93.02	S8*	91.48	96.91	S8**	90.05	95.39	S8**
**ABCDF Lingual**	59.3	77.03	S8***	68.35	85.22	S8***	70.7	81.29	S8**
**Mesial**	78.12	68.71	S7***	69.68	83.54	S8***	68.87	74.02	S8*
**Distal**	78.1	64.94	S7***	66.47	82.21	S8**	63.71	71.9	S8**
**Total**	71.37	68.81	S7*	66.47	81.29	S8**	66.95	73.65	S8**
**Hardness (S2 vs. S4)**									
	**Horizontal**			**Rotating**			**Vertical**		
	**S2**	**S4**	**p-value**	**S2**	**S4**	**p-value**	**S2**	**S4**	**p-value**
**Buccal**	94.37	96.62	S4*	94.76	96.73	S4**	80.83	79.46	NS
**ABCDF Buccal**	61.16	72.61	S4***	58.91	72.96	S4***	74.72	74.27	NS
**Lingual**	95.96	97.5	S4**	95.81	97.32	S4**	94.12	93.11	NS
**ABCDF Lingual**	77.8	86.58	S4**	77.44	86.19	S4***	79.17	80.85	NS
**Mesial**	69.28	76.5	S4*	60.5	72.96	S4**	67.99	68.74	NS
**Distal**	68.74	74.72	S4*	58.79	66.32	S4***	58.3	58.98	NS
**Total**	68.11	75.66	S4**	62.33	72.63	S4*	67.94	68.67	NS
**Hardness (S2 vs. S6)**									
	**Horizontal**			**Rotating**			**Vertical**		
	**S2**	**S6**	**p-value**	**S2**	**S6**	**p-value**	**S2**	**S6**	**p-value**
**Buccal**	94.37	92.24	NS	94.76	92.85	NS	80.83	79.91	NS
**ABCDF Buccal**	61.16	53.87	NS	58.91	62.45	NS	74.72	55.5	S2***
**Lingual**	95.96	93.98	S2**	95.81	93.78	S6**	94.12	91.51	S2*
**ABCDF Lingual**	77.8	69.05	S2*	77.44	73.87	S2***	79.17	70.44	NS
**Mesial**	69.28	88.68	S6***	60.5	82.38	S6***	67.99	70.34	NS
**Distal**	68.74	87.32	S6***	58.79	76.85	S6***	58.3	70.47	S6***
**Total**	68.11	80.31	S6**	62.33	77.32	S6***	67.94	68.92	NS
**Hardness (S4 vs. S6)**									
	**Horizontal**			**Rotating**			**Vertical**		
	**S4**	**S6**	**p-value**	**S4**	**S6**	**p-value**	**S4**	**S6**	**p-value**
**Buccal**	96.62	92.24	S4*	96.73	92.85	S4**	79.46	79.91	NS
**ABCDF Buccal**	72.61	53.87	S4**	72.96	62.45	S4**	74.27	55.5	S4***
**Lingual**	97.5	93.98	S4**	97.32	93.78	S4**	93.11	91.51	NS
**ABCDF Lingual**	86.58	69.05	S4***	86.19	73.87	S4**	80.85	70.44	S4**
**Mesial**	76.5	88.68	S6**	72.96	82.38	S6***	68.74	70.34	NS
**Distal**	74.72	87.32	S6***	66.32	76.85	S6***	58.98	70.47	S6**
**Total**	75.66	80.31	NS	72.63	77.32	S6*	68.67	68.92	NS
**Interdent Height**									
	**Horizontal**			**Rotating**			**Vertical**		
	**S7**	**S8**	**p-value**	**S7**	**S8**	**p-value**	**S7**	**S8**	**p-value**
**Buccal**	85.32	80.03	S7**	88.04	96.54	S8*	76.94	95.39	S8*
**ABCDF Buccal**	46.94	68.05	S8***	53.64	75.32	S8***	63.35	70.37	S8**
**Lingual**	90.48	93.02	S8*	91.48	96.91	S8**	90.05	95.39	S8**
**ABCDF Lingual**	59.3	77.03	S8***	68.35	85.22	S8***	70.7	81.29	S8**
**Mesial**	78.12	68.71	S7***	69.68	83.54	S8***	68.87	74.02	S8*
**Distal**	78.1	64.94	S7***	66.47	82.21	S8**	63.71	71.9	S8**
**Total**	71.37	68.81	S7*	66.47	81.29	S8**	66.95	73.65	S8**

Table 1: Significantly higher percentage of plaque removal with each brushing movement using toothbrush pair indicated as: *p < 0.05; **p < 0.01; ***p < 0.0001; NS: No significant differences in any movement

### Head size

The comparison between the head-size of manual brushes S1 vs. S2 have shown in Table 1. For horizontal movements, S1 (Compact brush) have shown greater extent of simulated plaque removal than S2 (full head size brush) in four of the seven tooth fields (total area score, 82.42% vs. 77.88%) and the result was statistically significant (p < 0.05). For vertical movements, S2 demonstrated greater extent of simulated plaque removal in five of the seven fields than S1 (total area score, 75.86% vs. 70.91%), and the difference was statistically significant (p < 0.05). A significant difference was observed towards S1 in terms of horizontal and vertical movements than S2. However, for rotating movements, both brushes have shown significantly similar extent of simulated plaque removal.

### Filament diameter

On comparing simulated plaque removal based on differing filament diameters for S3 (extra soft brush) and S4 (soft brush) during horizontal movements, S3 demonstrated significantly better in simulated plaque removal in six of the seven fields than S4 (total area score, 86.74% vs. 75.66; p < 0.05%). S3 also led to statistically significantly superior results (p < 0.01) in rotational movement as compared to S4 (total area score, 79.98% vs. 72.63%). Additionally, both brushes have shown similar range of simulated plaque removal with vertical movements in all fields apart from distal field (total area score, 69.59% vs. 68.67%). Based on brushing movements, filament diameter comparison showed a significant difference favouring extra soft brush (S3) efficiently removing simulated plaque than soft brush (S4) with measurable statistical differences (Table 1).

### Cutting height

When comparing simulated plaque removal with S2 (larger cutting height-12 mm brush) and S5 (smaller cutting height 9 mm brush), In horizontal movements, S2 exhibited significantly greater extent of simulated plaque removal in all fields as compared to S5 (total area score, 68.11% vs. 47.22, p < 0.01%). In rotating movements, only four fields have shown better simulated plaque removal favouring S2 as compared to S5 (total area score, 62.33% vs. 57.96%). The difference in rotational movement was statistically significant (p < 0.05). For vertical movements, four of the fields showed significantly better simulated plaque removal with S2 (total area score, 67.94% vs. 51.57%) as compared to S5, although in two of the fields (Lingual and Buccal) S5 brush have also shown significant advances. The larger cutting height brush (S2) was better in removing simulated plaque which was statistically significant when compared to smaller cutting height brush, S5 (p < 0.05; Table 1).

### Hardness

Brushes of differing hardness included the medium hard S2 brush, the soft S4 brush and the soft S6 brush (Table 1). Compared to S2, the softer S4 brush removed a higher percentage of simulated plaque in all tooth fields with both horizontal (total area score, 68.11% vs. 75.66%) and rotating (total area score, 62.33% vs. 72.63%) movements. No differences were found for vertical movements (total area score, 67.94% vs. 68.67%). Overall, looking at the total area score in horizontal movements, S6 exhibited better simulated plaque removal in two of the fields (total area score, 68.11% vs. 80.31%) and S2 demonstarted better simulated plaque removal in 4 fields as compared to S6. But the results for S2 was significant in 2 fields only and other two were non significant when compared to S6.

For rotating movements, the softer S6 was significantly more efficient overall and in three of the tooth fields with no advantages for the medium hard S2 brush (total area score, 62.33% vs. 77.32%). For vertical movements, it was the the S2 brush that was more efficient, in three of the areas compared to one for S6, but no advantage was shown for the Total areas (total area score, 67.947% vs. 68.92%). The difference was statistically significant (p < 0.05), favoring the simulated plaque removal efficacy with soft S6 brush.

Finally, when the two soft brushes were compared, the S4 brush was significantly better in some areas (Buccal and Lingual, except vertical movement, ABCDF Buccal and ABCDF Lingual, all movements), but the S6 brush was better in Mesial and Distal areas. However, there was little difference when examing Total area, with a significance difference (total area score, 72.63% vs. 77.32%) only shown for the S6 brush in the rotating movement (Table 1).

### Interdental height difference

For the horizontal movements, S7 interdental brush with interdental cut of 10/11 mm demonstrated better simulated plaque removal efficacy as compared to S8 toothbrush with a larger interdental cut of 8.5/11 mm (total area score, 71.37% vs. 68.81%). The difference in some areas i.e. Buccal, Mesial, Distal, Total reached a statistical significance (p < 0.05). However, in other areas (ABCDF Buccal, Lingual, ABCDF Lingual), S8 demonstrated a significantly better performance in removing interdental simulated plaque compared to S7. For rotating and vertical movements, the S8 brush (with larger interdental cut) removed statistically higher percentage of simulated plaque interdentally in all areas including total areas as compared to S7 brush (rotational: 81.29% vs. 66.47%; vertical: 73.65% vs. 66.95%; p < 0.01; Table 1).

The toothbrushes displayed no deformation of bristles following the testing.

## Discussion

Mechanical plaque removal by regular tooth brushing facilitates the prevention of plaque-associated oral diseases and their sequelae [14]. There are a wide range of toothbrushes available on the market with different designs and claims related to cleaning efficacy [15]. Laboratory testing of different toothbrush design parameters for their cleaning efficacy is essential for the development of new prototypes. However, statistically significant differences shown in vitro due to variations in the configuration of the tufts should be interpreted with caution when inferring clinical implications. Generally, normal brushing consists of a mixture of horizontal, vertical and/or circular scrubbing movements, which can vary from person to person [16]. Any laboratory testing should therefore, be as close as possible to the real clinical conditions [10]. From a bio-physical point of view, the reproduction of random brushing movements in the current study were best achieved by separating the dynamic process of tooth cleaning into these three basic movements. Thus, this study was designed to evaluate five different bristle parameters (head-size, cutting-height, hardness, filament diameter and interdental-height difference) to assess the extent of simulated plaque removal efficacy in a clinically validated robot-based setting using horizontal, rotating and vertical movements.

Studies in literature have suggested that the toothbrush design parameters along with technique and movements of brushing largely impacts the effective plaque removal [17]. Likewise, results of the present study also demonstrated that different toothbrush parameters and movements significantly influence the level of simulated plaque removal. The circular movements during the rotating brushing action, as used in modified bass technique of tooth-brushing, are considered to be the best [16, 18]. These are also used in a number of commercially available power toothbrushes as reflected in their oscillating-rotating movements [19]. Thus, this forms an important movement to be assessed. In the present study, the simulated plaque removal was most efficient with rotating movements as inferred from the total area scores for all design parameters. From the results of total area scores in present study, for rotating movements only, the best performing toothbrush would be the one with a compact head as opposed to a full head, a smaller filament diameter (0.12 mm compared to 0.15 mm in this study), a larger tuft height (12 mm compared to 9 mm), a soft brush (0.15 or 0.18 mm filament diameter compared to 0.23 mm,) and a greater difference between tuft heights (8.5 and 11.0 mm compared to 10 and 11 mm). For Total Area Scores, most of these variables were also better in horizontal movements and, in general, results for individual areas also concluded the same. However, there were fewer differences when examining vertical brushing movements.

Interestingly, for the toothbrush characteristic of hardness; medium hard S2 brush vs. the soft S4 brush; there was identifiably one brush more efficient than the other in all areas. The results of the study were inconclusive for the head size of manual toothbrushes, S1 vs. S2 (compact Vs full head size). S2, full head size was significantly more effective than S1, compact brush in vertical movements (p < 0.05), whereas it was vice-versa in horizontal movements and similar simulated plaque removal with both brushes was observed in rotating movements. This shows how brushing movements may be important when instructing on most efficient toothbrushing method. It also highlights that while the rotating movements of the Modified Bass Method may be considered better for general oral healthcare instructions [16, 18] for people with problems due to plaque accumulation in specific tooth areas, adding horizontal and/or vertical movements may be of benefit, depending on the toothbrush used and taking into account factors such as dental erosion where over aggressive horizontal movements may be detrimental [20–22].

Yankell et al., in their study comparing 2 manual toothbrushes with a laboratory method also demonstrated tapered bristles to be more effective in comparison to the brushes with rounded bristles in a flat-head [23]. From the present study also, it could be inferred that the toothbrush with more compact head, longer and softer filaments of differing lengths may allow filaments to flex more while tooth brushing, and reach more tooth-surface area resulting in better and effective plaque removal.

To the authors’ best knowledge, in English literature, this study was the first to assess the efficacy of all given toothbrush design parameters with different brushing movements in a single study using robot-based setting and plaque removal calculations by APP.

A limitation of this study is that it was in vitro with a robot carrying out the movements. For these findings to be used to help educate patients on the best brush design for them, complementary in vivo studies using clinical plaque indices and/or planimetrical plaque indices and blinded photographic assessment need to be carried out to understand how each movement and toothbrush characteristic affects plaque removal.

## Conclusion

This robot brushing in vitro study showed that overall, filaments that were longer, softer and/or had a difference between filament length showed greater simulated plaque removal compared to short and hard filaments as would be found in a standard, flat trim, stiff toothbrush with a larger head. This study could provide further insights, leading to improvements in toothbrush design and performance. However further studies are needed to assess clinical differences between the toothbrush parameters.

## Data Availability

The complete datasets generated and/or analysed in the current study are not available publicly in the manuscript due to their lack of relevance to this article and word count considerations. Although, they are available from the corresponding author on reasonable request.

## Abbreviations

APP: Automated plaque planimetry
3D: Three dimensional
WMW: Wilcoxon–Mann–Whitney
